# English as a Foreign Language Teachers’ Well-Being, Their Apprehension, and Stress: The Mediating Role of Hope and Optimism

**DOI:** 10.3389/fpsyg.2022.855282

**Published:** 2022-03-15

**Authors:** Hui-min Yang

**Affiliations:** Department of Basic Teaching, Jiangsu Food and Pharmaceutical Science College, Huai’an, China

**Keywords:** apprehension and stress, teachers’ well-being, EFL teacher, hope, optimism

## Abstract

Studies have shown that teachers’ wellbeing has a positive effect on teachers’ learning quality and learners’ performance. Nevertheless, teaching is a stressful and exhausting profession at all academic level with special difficulties about the nature of language education. Tension and fear are still classic challenges in learning, though the concepts such as hope and optimism are core issues in assisting teachers to feel happy during instruction and work longer. The present review makes efforts to provide the most current confirmation on the interface of hope and optimism with educational issues since they are progressively documented as significant emotional capitals for educational success, job growth, and presentation. It is worth mentioning that the current review of research can benefit educational administrations, and other stakeholders and officials in the educational community to contemplate the functions of constructive emotions in the process of learning to decrease and even diminish stress and apprehension that consequently lead to flourishing.

## Introduction

The place of work can be at the heart of stress and psychological health issues as a result of problems such as great degrees of emotive or intellectual needs, the absence of chances, work-related burden, or feeling underappreciated (Eurofond, 2012; Beehr, 2014). Teaching is perceived as a difficult and stressful career as it requires learners’ trust to form themselves in the educational system and it is revealed that facing numerous challenges will put educators at risk of quitting or burnout (Ghanizadeh and Jahedizadeh, 2015). Stress or tension is viewed as a negative affective experience triggered by teachers’ perceptions that their professional conditions pose a threat to their happiness and wellbeing (Kyriacou, 2011). Undoubtedly, stress has been regarded as a deconstructive encounter that jeopardizes both educators’ self-confidence and wellbeing and hinders the educational cycle (Klassen and Durksen, 2014). Particularly, foreign language teachers can be subject to additional burdens and tension caused by the difficulties of helping foreign language students master their competence (Shah et al., 2013). The stress in teachers can leave overwhelming significances for both teachers and the value of teaching. Potential concerns of teacher stress decrease self-efficacy, decrease gratification of job, decrease the level of involvement, increase burnout, and improve attrition (Collie et al., 2012; Klassen et al., 2013). Concerning the recent literature, teaching is a distressing task in many nations across the globe, and educator stress is certainly a universal occurrence (Skaalvik and Skaalvik, 2016). Also, specified that instruction is regarded as one of the most distressing careers in which most educators experience utmost stress because of the inherent attributes of the career. Stress is an intricate topic among educators and is well-reported in the literature (Malik and Ajmal, 2010). One of the influential factors of burnout and attrition in teachers relates to the emotive aspects of education, predominantly the anxiety about various occupation facets (Ghanizadeh and Ghonsooly, 2014). As Horwitz (2017) proposed, an individual sense of tension, apprehension, and anxiety altogether with the stimulation of the autonomic nervous scheme is called anxiety. Within interactive jobs like teaching, the part of anxiety called apprehension that seems a more appropriate concept. As stated by Kyriacou (2011), apprehension is theorized as situation-related anxiety regarding the future, particularly dealing with unpleasant and difficult things. The prediction of troublesome or pessimistic occurrences is one of the hallmarks of apprehension. Another characteristic of apprehension is that it is strongly associated with fright or nervousness that accompanies communication (Li, 2021).

Rather than highlighting avoidance or reduction of negative aspects like stress, fear, and anxiety that are associated with second language learning, reinforcing positive aspects against external threats can lead to a successful outcome in language learning, thereby contributing to competence development when coping with and controlling everyday tension and frustration (Oxford, 2016). Alternatively, PP aims at improving the excellence of life rather than solving the current difficulties (Seligman, 2011) and also it pays attention to the appropriate concentration of competencies and abilities, rather than difficulties (Kurz, 2006). Positive psychology is a collective term that includes wellbeing, pleasure, gratification, optimism, beliefs, and enthusiasm for work which are related to particular constructive involvements (Dewaele et al., 2019; MacIntyre et al., 2019). Wellbeing is the main notion explored in the field of PP and the emphasis of PP is on aspects contributing to helping tutors and students have fun with instruction and education practices and sustain great degrees of wellbeing (Wang et al., 2021). It is a multidimensional hypothesis that involves constructive feelings, joy, involvement, constructive associations, significance, and accomplishment or success (Seligman, 2011; Seligman and Csikszentmihalyi, 2014). As a result, understanding teachers’ psychology is needed since their mindsets and professional wellbeing are connected to their teaching quality along with learner performance (Mercer, 2018). Educators with positive mind states are willing to be interested in their occupations and display higher inspiration and improved teaching skills.

In this context, wellbeing is characterized as the accrediting result that can enable both individual and social changes. Seligman’s wellbeing hypothesis emphasized constructive feelings that expand a person’s chances and build their abilities and skills (Fredrickson, 2004; Seligman, 2011). In line with Seligman (2011), the goal of the wellbeing hypothesis was to advance prosperity by enhancing constructing feelings, involvement, significance, constructive connections, and achievements. Consequently, there is rising interest in studying protecting features and interventions encouraging wellbeing in the workplace (Shropshire and Kadlec, 2015). For instance, individual psychological traits such as high degrees of self-efficacy, optimism, hope, self-confidence, and resilience significantly can be effective (MacIntyre et al., 2019; Jin et al., 2021).

The significance of optimism can be justified by PP, which concentrates on individuals’ strengths and positive personal qualities, as well as their promotion for humans (Seligman and Csikszentmihalyi, 2014). Indeed, a rather recent notion in educational psychology is academic optimism that is formed by integrating the three intellectual (self-effectiveness), emotional (faith in learners and guardians), and developmental (focus and urging scholastic) factors. Roused by PP, it makes up a strong group of principles that could be utilized as a suitable measure to foresee or evaluate the performance of educators in career life, psychological wellbeing, and psychological health (Beard et al., 2010). Having been stimulated by PP, it creates an influential set of beliefs and principles that may be implemented as an appropriate guide to guess or evaluate the personal presentation of educators in specialized life, and wellbeing (Fluck and Dowden, 2013). Academic optimism is a constructive conviction in educators dictating that they are capable of triggering learners’ scholastic success with an accentuation on instructing and education, with students’ involvement, with confidence in their capability to conquer hardships and failures, and with thorough endeavors (Hoy et al., 2006).

Moreover, the notion of hope may be clear to many; however, the technical conceptualization of hope started with hypotheses of hope in modern times (Gallagher et al., 2018), and in specific, in the domain of PP (Snyder, 2000; Seligman, 2006). As stated by Snyder (2000), hope is considered as a notion associated with optimism, within the context of the capability of scheme routes to attain an objective that the person seeks despite the existence of impediments and an inspirational origin to use these routes. Hope is characterized in this context as a resolution to reach an objective and a conviction that there are numerous other methods (Hefferon and Boniwell, 2011). Indeed, hope is the strongest when it is based on the beneficial aims that have medium chances of attainment and are acquired from difficult but not impenetrable hindrances. Thus, a person does not require hope when he or she is confident in attaining objectives, and he or she becomes distressed when thoughts of not succeeding come to mind (Carr, 2013). As Bashant (2016) declared, hope is the impetus for feelings and wellbeing which are explained as crucial elements of a person’s enjoyment and achievement in life. The method addressing optimism in the milieu of attribution methods and the hope theory in which Snyder formed the idea of hope as energy striving for significant objectives, indicates that people can alter their levels of optimism and positive expectations in a significant manner. Therefore, people can stay optimistic and maintain their hopes by determining objectives and possessing constructive thoughts regarding attaining those objectives, sustaining inspiration to prepare schemes for achieving those objectives, and sensing the involvement in these cycles that will facilitate the attainment of those objectives. Studies carried out to propose that hope is an element closely associated with the degree of a person’s wellbeing (Dursun, 2012).

The great number of studies (Travers, 2001; Kyriacou, 2011), on the topic of career stress in general, and educator stress in specific, are signals that the problem is highly significant for researchers across the globe that requires more accentuation. Introducing educators’ stress as a critical point, educators with a great degree of stress represent a lower degree of career engagement (Jepson and Forrest, 2006). Deconstructive influences of stress on educators involve lessened self-effectiveness, drained educator-learner connections, exhaustion, and greater frequencies of educator absenteeism and educator turnover (Harris, 2011; Klassen et al., 2013), in addition to decreased self-esteem, blocked scholastic objectives, and elevated chances of giving up (Leroux and Théorêt, 2014). Therefore, to assist with lowering stress and elevating wellbeing for educators, Greenberg et al. (2016) have emphasized the need to focus more on institutional and personal interferences.

Based on the aforementioned studies conducted so far, the effect of teachers’ stress and apprehension on the one hand and the educators’ hope and optimism, on the other hand, are all well-proven in the literature; nevertheless, little is acknowledged about the role of these issues on teachers’ wellbeing in the classroom. For that reason, this paper attempts to enhance the prior literature regarding these concepts altogether in language teaching.

## Background

### Stress

Stress has been characterized as an element that functions inside or outside and makes adjustment difficult; therefore, more endeavors are required from the person to sustain balance in himself or herself and from the outer setting (Abebe and HaileMariam, 2011). Moreover, Educator stress may be characterized as an educator’s encounter of displeasing, deconstructive feelings like rage, stress, pressure, exasperation, or sadness as a consequence of some dimension of their career as an educator (Kyriacou, 2011). There are two primary kinds of inconveniences: individual and contextual. Individual embarrassments involve thoughts, convictions, and inner emotions that make a person’s role difficult, while contextual inconveniences involve certain events like when an educator observes a dispute between learners. Inconveniences are ubiquitous, but the various manners in which people respond to stress means that the stress reaction is not global because one individual can manage inner stress better than another (Xin et al., 2017; Rajabi and Ghezelsefloo, 2020).

In addition, numerous stressors exist that occur in the teaching field, such as internal stressors. Internal stressors increase when teachers lack communication skills, when teachers feel lonely without peer help, and when teachers are supervised (Kyriacou, 2011). When teachers are concerned about building good relationships with learners external stressors are activated when teachers are not updated about instructional materials and learning instruments, when teachers have to handle time limit to teach the study subjects to rest during the breaks, when teachers have a busy schedule with too much work, or when they must deal with unmotivated or absent learners (Kyriacou, 2011).

### Apprehension

Apprehension influences the goals of an individual, profession, lifestyle, demeanor, and even identities (Kyriacou, 2011). The anticipation of unpleasant or adverse events is the unique characteristic of apprehension. The other unique characteristic of apprehension is that it is closely related to fear or nervousness when communicating with others because it is used synonymously with the apprehension of communication (Kyriacou, 2011). Teaching a foreign language is frequently stressful for many people, especially in class which is just like when anxiety avoids individuals from succeeding in science or math. Thus, it is a common or inclusive term that encompasses all areas and atmospheres (Kim and Kim, 2004). Apprehension refers to a physical and emotional response to an expected risk or condition with intellectual, emotional, and control facets and features that influence the ability to contemplate and process information (Malik et al., 2020). Such intellectual section interferes with educational and communication progressions which also influences thought processes (Malik et al., 2020).

### Optimism

As stated, Constructive anticipation of what is to come is known as optimism, and it portrays a condition of awareness, feeling, and inspiration for the future. Future beliefs have a strong influence on present tendencies, and attainable objectives influence a person’s behavior. Optimistic individuals concentrate on the positive aspects of life and have faith that they can continue to have aspirations in hard times and conquer such difficulties (Schueller and Seligman, 2008). Academic optimism is different from scholastic optimism; the latter (for example, optimism for life) alludes to individual inclinations like overall demeanors and a constructive viewpoint regarding the future; the former is particular to instruction and education (Beard and Hoy, 2010). Hoy et al. (2006) coined academic optimism, and it is a newer framework than scholastic optimism. Academic optimism involves scholastic self-effectiveness, faith, and scholastic importance at the personal and institutional level (Hoy and Tarter, 2011). Research suggests optimism as an important predictor of academic performance and psychological well-being (Rand et al., 2020).

### Hope

Snyder theorized hope as constructive anticipation regarding what is to come dependent on a dual capability of creating particular routes to objectives and having the individual means to execute those routes to effectively attain one’s objectives in line with former achievements, current will, and future eagerness (Snyder, 2002). When a person with a high level of hope encounters difficulty, he will come up with a route to the objective, possess the means to execute the route, and then change the route and try again if the first endeavor was insufficient. Individuals with ample hope believe they will achieve their objectives. Moreover, they regard hard objectives as difficulties they can overcome as opposed to risks that must be evaded. Hope is frequently associated with better psychotherapy results, adjustive management, emotive wellbeing, physical fitness, and education (Cheavens et al., 2005; Gallagher et al., 2018).

In reference to goal-directed thinking, hope involves the capability of conceptualizing objectives, evolving tactics to achieve them, and sustaining sustainability in using those tactics to accomplish objectives (Marques et al., 2014). Also, people with high hopes are willing to have a stronger path and more thinking persistence to achieve their objectives than people with low hopes (Snyder, 2002; Marques et al., 2014). Studies have demonstrated that hope has an important and constructive connection with predictors of wellbeing, namely, universal life fulfillment and psychological health, and it is pliable to alterations through interferences that elevate a person’s objective determining manners (Marques et al., 2014). Snyder’s hypothesis on hope involves several elements, namely, deciding on achievable objectives, possessing the inspiration and persistence needed to seek these objectives, and ultimately, achieving these objectives (Snyder, 2002). Through achieving objectives, the dexterity attained by learners inspires them to go through the process again, thereby resulting in more achievements (Bashant, 2016). The classroom setting is a significant issue in providing the circumstances most encouraging to hope.

### Well-Being

One rationale for the enhancement of wellbeing is that it involves more than feeling well and individuals who are satisfied with their lives are more effective and more engaged (Peterson et al., 2006). Those who reported great degrees of wellbeing experience constructive feelings more often by learning assignments that are regarded as an evaluation and judgment of the entire educational cycle (Jing and Yu, 2015; Yuting et al., 2020). The perception of wellbeing was described by Seligman (2011) in five main dimensions, all of which fall into the constructive continuity of mental wellbeing identified as constructive feelings, commitment, relations, meaning, and achievement (PERMA). Constructive emotions such as enjoyment, optimism, and wellbeing are included under hedonic continuity of emotive conditions that work as signals of flourish, although they can assist an individual with prospering and can be instructed and enhanced (Fredrickson, 2001). A motion or profound involvement aimed at being inspired naturally while completing an assignment is known as engagement (Dixson et al., 2017; Derakhshan, 2021). Objective determination, monitoring, and achievement all add to wellbeing all through life (Heckhausen et al., 2010).

Positive relationships refer to the feeling of being societally involved, appreciated, and strengthened by others and being satisfied with a person’s societal connection. Societal help has been linked with constructive spiritual and physical wellbeing results and wellbeing, in general (Karademas, 2006; Greenier et al., 2021). Meaning alludes to the notion that a person’s life has persistence and a path in the lifespan, and to have a sense of attachment to something bigger than oneself. Meaning has been linked with wellbeing results as well as with constructive feelings in various age categories (Cotton Bronk et al., 2009). Achievement is normally connected to objective determination, development, and the capability of achieving, hence, endeavoring for wellbeing (Croom, 2015).

## Pedagogical Takeaways

Built on the conclusion taken from the review of related literature, several deductions and suggestions for policymakers, practitioners and researchers are provided. In the same vein, the current review has some practical suggestions for english as a foreign language (EFL) teachers, teacher trainers as they care about teachers and professionals in the EFL educational field to widely spread their outlook on the prominence of educators’ stress and apprehension and the factors related to them. Simply, the education career is stressful, because it inevitably entails unpredictable and unanticipated situations that can cause tension and stress.

Built on academic concerns and widespread preceding investigations, it is revealed that optimism qualifies teachers to handle ambiguity and demanding job procedures positively as optimist educators get involved in more dynamic managing procedures, look for more societal provision, put emphasis on the constructive facets of problematic and challenging circumstances, and are capable of regulating these traumatic circumstances in the process of their teaching. Teachers feeling high stress and apprehension with low coping capabilities are negatively connected to poor learner outcomes (Herman et al., 2018).

Furthermore, this review is crucial for educators in the language learning circumstance as they should be aware of their noteworthy role in this process. They are the main factor in any scholastic milieu specifically in language learning and their well-being should be central in the scholastic background to increase their learners’ aptitude and to stimulate them to participate in activities. Indeed, educators with great degrees of academic optimism have great scholastic objectives for their learners, try to instruct successfully in class to attain the determined goals, and have faith that every learner can attain success (McGuigan and Hoy, 2006). Educators can be anticipated to possess academic optimism in schools where managers assist educators, regard them as professionals in their discipline, make their work easier, and prove that they hold their capabilities, knowledge, and abilities in high regard.

Optimism offers an objective for mediations at the teacher-level as Hoy (2012) postulates that at this level, intellectually optimistic educators try to set objectives for themselves and for their learners that are detailed and stimulating, and the ones that care about success. This concept, optimism, is noteworthy for educators as it is maintained that there is a meaningful negative correlation between optimism and stress, meaning optimistic individuals are likely to have less hypothetical stress (Huan et al., 2006). Thus, the results offer basic support for the concept that organizations can improve their constructive responses to workforce changes by raising hope levels and improving positive communication with leaders through training or mentoring (Snyder, 2000).

Educators who have a great degree of hope are normally capable of expressing various ways of attaining this objective that allows them to assist learners in perceiving their skills (Shade, 2001). The mediator role of hope in the association between teachers’ wellbeing and stress is maintained in the literature, based on it, the enhancement of hope in educators could also be valuable in teaching. Even though hope is a moderately steady and constant attribute of personality, there is a cumulative indicator that efforts to encourage hope can be efficacious (Gallagher et al., 2018). Hope in itself has been revealed to have a constructive function in diverse realms from accomplishment to wellbeing and is apparent in a developing body of literature (Peterson et al., 2006; Hartley et al., 2008). Therefore, the conclusion drawn from this review propose that educational personnel should thoroughly try to build educators’ hope, as a resource to expand their wellbeing. Likewise, hope is regarded as a superior mediator of wellbeing in comparison with parallel concepts such as optimism or efficacy as a result of its distinctive features against its corresponding item. The review also offers introductory provision for the notion that school managers might have beneficial reactions to the adjustment in their personnel through developing degrees of hope by training, teaching, and improving constructive connections with faculty members.

Furthermore, agencies and school administrators are supposed to shape the educational system in a way that supports teachers in a collaborative and well-equipped environment. Teacher trainers are encouraged to help teachers have education programs that increase awareness about work-related anxiety and the affective aspects of learning. Educators should also build a curriculum that finds debilitating fear and enables teachers to change them into those with tactical and regulatory functions. Based on expectations, such programs give teachers the courage to overcome tension and unmask their sense of superiority in class to prevent their unpleasant feeling.

The school faculty members should try to increase teachers’ hope in the process of their working as it is emphasized by Park et al. (2004) that hopeful people are inclined to possess a constructive perspective regarding their future, giving them convictions enabling them to possess a constructive viewpoint regarding themselves that helps in elevating their inspiration and results in culminate actions and paths oriented toward actively seeking individual objectives. Therefore, hopeful people have a higher chance of succeeding in their attempts and this gives them a feeling of satisfaction which elevates their wellbeing. The review of literature provides introductory provision for the indication that faculty members might improve constructive reactions to adjust in their staff by growing levels of hope, through training or mentoring, and through enhancing positive relationships with faculty members (Snyder, 2000). In this study, the PP factors were hope, optimism, and wellbeing, more studies must highlight various theories concerning constructive tendencies involving resilience and perseverance as indicators of various issues in PP and must examine their significant effect on language education.

## Ethics Statement

The studies involving human participants were reviewed and approved by Jiangsu Food and Pharmaceutical Science College Academic Ethics Committee. The patients/participants provided their written informed consent to participate in this study.

## Author Contributions

The author confirms being the sole contributor of this work and has approved it for publication.

## Conflict of Interest

The author declares that the research was conducted in the absence of any commercial or financial relationships that could be construed as a potential conflict of interest.

## Publisher’s Note

All claims expressed in this article are solely those of the authors and do not necessarily represent those of their affiliated organizations, or those of the publisher, the editors and the reviewers. Any product that may be evaluated in this article, or claim that may be made by its manufacturer, is not guaranteed or endorsed by the publisher.
